# Mitrofanoff procedure in children: use of the appendix and VQZ plasty seems to minimize complications

**DOI:** 10.1007/s00383-025-06204-6

**Published:** 2025-09-26

**Authors:** Adriana König, Ashley X. Wiseman, Barbara Wildhaber, Isabelle Vidal, Jacques Birraux

**Affiliations:** University Center of Pediatric Surgery of Western Switzerland, Division of Child and Adolescent Surgery, Department of Women, Child and Adolescent, University Hospital of Geneva, Geneva, Switzerland

**Keywords:** Mitrofanoff, Continent catheterizable channel, Long term follow-up, Children

## Abstract

**Purpose:**

Variations around Mitrofanoff technique exist for continent catheterizable channel (CCC). The present study aims to analyze CCC complications within our patient cohort, in which the appendix was given preference to the maximum.

**Methods:**

Retrospective review of pediatric patients who had CCC surgery in our institution (2007–2019). A nine-item questionnaire was sent to assess current use of their CCC.

**Results:**

Among the 31 patient-cohort, appendix was used in 30, with a VQZ plasty in the right lower quadrant in 29. Postoperatively, seven CCC dysfunctions occurred in six patients between one month and nine years (four catheterization difficulties, one CCC stenosis at bladder level, one CCC incontinence, one parastomal hernia, no superficial stenosis). Of these complications, two were managed conservatively, two endoscopically, and three by open surgery. 21/23 surveys were returned: mean age of responders was 15 years, 97% used their CCC regularly, and none complained of CCC incontinence.

**Conclusion:**

This study shows a low revision rate for CCC dysfunction, with over half of the cases effectively managed through conservative or endoscopic means. It seems that following a stringent protocol with meticulous surgical technique and standardized postoperative care by a specialized nursing team reduces the risk of CCC complications.

**Supplementary Information:**

The online version contains supplementary material available at 10.1007/s00383-025-06204-6.

## Introduction

Adequate bladder function requires complete voiding in a low-pressure system, protecting the upper urinary tract. Some diseases result in bladder dysfunction, with not only impaired renal function but also socially disabling incontinence. In the 1970 s and 80 s, concepts developed by Lapides and Mitrofanoff drastically changed the management of patients with severe bladder impairment. In 1972, Lapides introduced the concept of clean intermittent catheterization (CIC), facilitating bladder emptying by self-catheterization through the urethra [1]. This allows for controlled voiding and social continence of up to three hours [2–4]. Some patients, however, struggle with trans-urethral catheterizations. Main reasons are intact urethral sensibility or physical disability requiring catheterization to be performed by another person [5, 6]. For the latter, manipulation of genital organs can be a psychological burden, as well as technically difficult if the patient is, for example, wheelchair bound [6]. Hence, another milestone was achieved by Mitrofanoff in 1980, publishing his concept of a continent catheterizable vesicostomy [7], allowing for a much easier catheterization, overcoming the aforementioned obstacles.

Since its first description, the Mitrofanoff technique has undergone numerous modifications, such as type of conduit, localization of the cutaneous stoma as well as its technique for creation [8]. Mitrofanoff initially used the appendix as conduit [7], but nowadays, the term “Mitrofanoff principle” commonly refers to any continent catheterizable channel (CCC). The cutaneous stoma can be placed in the right lower quadrant or in the umbilicus. Different techniques have been described for its anastomosis at the skin level, coming from direct suturing to diverse skin flaps [9, 10].

Even though the concept of a CCC has been universally adopted [11], outcomes show a high percentage of complications, ranging from 20 to 60% [12–16]. Most common CCC complications are stomal stenosis at skin level and stomal urinary leakage [2, 4, 9, 12, 17, 18]. Complications seem to occur at two time points: either soon after initial surgery [9, 18–20] or after several years of using the channel, consistent with the effect of long-term conduit use [6, 20–23]. Complications have been reported to often require reoperations [9, 12, 22].

A comprehensive review of existing literature does not conclusively determine which among the various conduit types, skin anastomosis techniques, and locations is associated with the lowest complication rates [8, 16, 20, 21, 24]. More data is needed. Thus, the present study aims primarily to analyze CCC complications within our patient cohort in which the appendix was given preference to the maximum, focusing on the timing of their occurrence and the necessity for surgical or endoscopic revisions.

## Materials and methods

Patients having undergone a CCC procedure in our institution, aged 0 to 18 years, between 01/01/2007 and 31/12/2019 were included. They were identified using (1) our hospital’s operating room documentation system, and (2) personal notes from the senior author (JB). We excluded patients with a follow-up (FU) period of less than twelve months and if patients or their legal guardians refused to participate in the study.

*Variables:* Data were collected from the patients’ medical records. Included variables were the following: patient age at intervention, underlying medical condition, reasons for CIC and indication for CCC surgery; additional interventions at the time of primary surgery and surgical details; every additional intervention after CCC surgery; complications after CCC surgery. Postoperative complications were stratified in (1) dysfunction of the channel (stenosis, leakage, difficulties in passing a catheter, any other condition that would impair its use) and (2) other.

*Surgical and FU protocol:* During the entire study period, a standardized protocol for CCC surgical technique and patient FU was used. Whenever possible, the appendix is used as conduit. The bladder anastomosis is placed in the lower part of the posterior bladder wall, creating an anti-reflux mechanism. Anteriorly, the bladder is hitched to the abdominal wall. At skin level, the conduit is preferentially anastomosed in the right lower quadrant with a VQZ-plasty, or if not possible, in the umbilicus with a V-plasty. If deemed necessary, bladder augmentation is performed during the same operation. Two senior staff members performed the surgeries on all patients. Perioperatively, antibiotics are administered for 5 days. All children receive anticholinergic treatment postoperatively. A supra-pubic catheter and a catheter in the conduit are kept in for one month. Patients are discharged on post-operative day 10 and re-hospitalized at day 30 to learn CIC via the new conduit. The first ultrasound is performed at three months post-surgery, repeated at six months, along with an urodynamic study and renal function monitoring. This is repeated at least once a year until transition to adult urology at the age of 16 to 18 years.

To gain additional information, we designed a nine-question data collection tool (additional data are given in Online Resource), which in 2021 was sent to all living patients and/or parents/legal guardians to investigate CCC utilization, details of CIC, current bladder medications, surgical revisions that may have occurred elsewhere, and any pregnancies/childbirth.

*Educational therapy:* All children are followed up by specialized urology nurses, trained in educational therapy, accompanying them before, during, and after hospitalization. At the beginning of the CIC discussion, children and their families meet with specialized nurses, who explain bladder function, bladder anatomy, and CIC principles. Explanations are customized to suit the patient’s comprehension level and are supplemented with educational tools such as videos, dolls, and drawings. If requested by the patient and the family, the specialized nurse may also organize meetings with other families in a similar situation. The same nurse will then accompany them during the perioperative stay and will teach them CIC through the conduit. Thereafter, the children are seen regularly by these nurses, who are also available if there are any urgent questions. The aim is to support families according to their needs, to help the children become more independent while accompanying them through the different stages of childhood and adolescence. Ideally, care is gradually transferred from the parents to the patient (Table 1).

**Table 1:** Roles of the urotherapy nurse (UN)

	Pre-operative preparatory training	CCC surgery	One-month post-operative	CIC in daily life	Adolescence to adulthood
Workplace	Out-patient clinic	In-patient clinic	In-patient clinic	Out-patient clinic	Out-patient clinic
Objectives	Tailored teaching Enhancing knowledge regarding urinary tract anatomy and physiology CIC-technical skills Evaluation of any misconception/fear/shame	Keep good communication with patient and family Coping with new CCC	Catheterization training protocol	Sharing of concerns with NP (phone contact) Motivation support Integration of CIC in daily life (school-hospital coordination, sport, etc.) Increase of autonomy and self confidence	Ensuring long-term compliance to CIC Evaluation of transition readiness
Tools	Drawings, dolls Mixed media-tools Handling of devices Meeting with other children performing easily their CIC	Dressing and medical care of the new CCC	Tell/show/perform-method	Adaptable follow-up Individual outpatient training at UN clinic	Transition assessment questionnaire
Actors	Multidisciplinary team: UN, psychologists, social worker, occupational therapist, and more if needed	Hospital medical team	Hospital medical team	Multidisciplinary team: UN, psychologists, social worker, occupational therapist, school nurses	Multidisciplinary team: UN, psychologists, social worker, occupational therapist, adult medical team
Timing	Step-by-step approach (3–5 sessions)	During peri-operative hospitalization	2 to 3 day-hospitalization	NP appointment combined with medical appointment	Transition consultations

*CCC* continent catheterizable channel, *CIC* clean intermittent catheterization

*Statistics:* Results are mainly descriptive, expressed as median, range, frequency, percentage.

*Ethical approval: *The study was approved by the Cantonal Research Ethics Committee (project ID 2020-01455). This research did not receive any specific grant from funding agencies in public, commercial, or not-for-profit sectors.

## Results

**Fig. 1:**
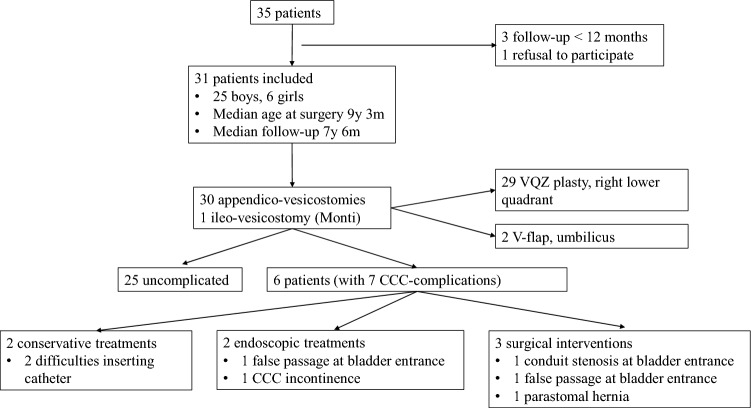
Cohort of patients with a continent catheterizable channel

Figure 1 describes the patient cohort: 31 patients were included in the study. At the time of our inquiries, two patients were deceased from unrelated causes but were still included, as time between surgery and death exceeded the minimal FU period.

Descriptive patient data is summarized in Table 2. At the time of channel creation, 21/31 patients underwent an additional intervention. Of those, ten were bladder augmentations. No patient needed a Malone antegrade continence enema procedure.

**Table 2:** Descriptive patient data

	Median (range)
**Median age at CCC intervention**	9.2 years (20 months—19.5 years)
**Median FU**	7.5 years (12 months—12.5 years)
**Initial diagnosis**	**Number of patients**
Neuropathic bladder	16
Sub-vesical obstruction:	
Posterior urethral valves	7
Other	2
Prune Belly Syndrome	1
Epispadias/exstrophy complex	3
Ano-rectal malformation	2
**Indications for CCC**	
Sensitive urethra	15
Difficulties regarding CIC handling because of impaired motricity	9
Both conditions	7
** Previous bladder augmentation**	0
**Type of conduit**	
Appendix	30
Detubularized segment of small bowel (Monti-tube)^a^	1
**Type of skin anastomosis**	
VQZ plasty in right lower quadrant	29
V flap in umbilicus^b^	2
**Additional interventions**^c^	
Bladder augmentation	10
Other	11

^a^Appendix of insufficient quality

^b^Insufficient length of the mesoappendix and/or suboptimal anatomical positioning

^c^No patient needed a Malone antegrade continence enema-procedure

### Continent catheterizable channel dysfunction and general complications

Seven CCC dysfunction occurred in 6 of the 31 patients, at 1 month to 9 years (5 with difficulties to pass the catheter, 1 incontinence through CCC, 1 parastomal hernia). Two patients encountered general complications (one intestinal obstruction post revision surgery and bladder augmentation, and one bladder stone). Figure 1 and Table 3 describe in detail CCC dysfunctions and general complications and their management.

**Table 3:** Postoperative complications: details of dysfunction of the continent catheterizable channel (CCC)

Patient no.	Underlying disease	Initial surgery (age at surgery)	Complication	Time after CCC	Reintervention	Clinical course
5	Posterior urethral valves	Mitrofanoff, bladder augmentation, renal transplantation (1 year 8 months)	Chemotherapy-associated mucositis causing difficulties inserting catheter (lymphoma 9 years after CCC)	9 years	*Conservative management*: Placement of indwelling catheter for 1 week	Recurrent mucositis with chemotherapy administration, otherwise uneventful
11	Malformation of the cervico-urethral junction	Mitrofanoff, bladder augmentation (6 years 2 months)	Difficulties associated with catheter insertion	1 month	*Conservative management*: Placement of indwelling catheter for 1 month	Uneventful for 7 years 5 months FU
10	Megalo-urethra, neonatal renal insufficiency	Mitrofanoff, nephrectomy, renal transplantation, bladder augmentation with ureteral patch (2 years 4 months)	Parastomal hernia	12 months	Superficial *surgical* stoma revision	Uneventful for 3 years 4 months FU
13	Posterior urethral valves	Mitrofanoff (2 years 6 months)	Asymptomatic chronic false passage of catheter at the appendix-bladder-anastomosis	20 months	Minimal *surgical* revision of appendiceal-bladder anastomosis, during enterocystoplasty for worsening chronic kidney failure	Uneventful for 4 years 6 months FU since endoscopic intervention
			False passage of catheter at the level of the bladder junction	6 years 7 months	*Endoscopic* modification appendicovesicostomy by deroofing of distal part of the conduit	
14	Posterior urethral valves	Mitrofanoff, bladder augmentation (15 years 5 months)	Urinary incontinence via CCC	3 years 7 months	*Endoscopic* Macroplastique® injection at appendiceal-bladder anastomosis	Uneventful for 7 years 3 months FU
15	Thoracolumbar myelomeningocele	Mitrofanoff (13 years 7 months)	Conduit stenosis at bladder wall level due to early displacement of indwelling catheter	1 month	*Surgical* revision of appendiceal-bladder anastomosis	Bowel obstruction unrelated to CCC one month postoperatively requiring laparotomy and adhesiolysis, uneventful FU of 9 years 2 months since

6 patients presented with 7 CCC-related complications

*FU* follow-up

No patient in our cohort required surgery for stomal stenosis at skin level.

Two patients presented with general postoperative complications without CCC dysfunction: one intestinal obstruction post revision surgery and bladder augmentation needing surgical lysis of adhesions, and one bladder stone needing surgical removal.

### Nine-question data collection tool

The questionnaire was sent to 23 families. Two of the 31 patients died of causes unrelated to their urologic surgeries, and 6 patients had changed address since last follow-up and were not reachable anymore. 21/23 patients returned the questionnaires (5/5 girls, 16/18 boys). Table 4 summarizes the results from the questionnaire.

**Table 4:** Results from the follow-up continent catheterizable channel (CCC)-questionnaire: 21 patients

	Median (range)
**Median age at questionnaire completion**	14.5 years (range 8.8—28 years)
**Median time from surgery**	8.6 years (range 2.3—11.5 years)
**Use of CCC**	
Daily	20/21
Stop^a^	1/21
**Catheterization**	
Frequency of catheterization	3–7 times/day
Size of catheter	12 or 14 French
Nocturnal catheterization	7/21
Anticholinergic medication	16/21
Urinary leakage from the CCC	0/21
**Continence**	
Complete continence	13/21
Occasional urinary stress incontinence	6/21
Significant urethral urinary incontinence	2/21^b^
**Pregnancy**	1/5 ^c^

^a^After renal transplantation in the context of posterior urethral valves, having regained almost normal bladder function

^b^One is satisfied with the use of pads and increased frequency of catheterization; the other is awaiting additional surgery;

^c^ uncomplicated pregnancy and vaginal delivery without conduit problems

## Discussion

This study reports on complications after CCC surgery in our patients, mainly operated with the appendix as a conduit and a VQZ plasty at skin level. The time of their occurrence and need for surgical or endoscopic revisions were evaluated. Of our 31 patients, 6 presented with CCC dysfunction, of which 4 needed surgical/endoscopic revision. These findings reveal a notably low complication rate compared to the existing literature. This strongly suggests the efficacy of our surgical and nursing protocols.

Of note, our pediatric patient population is comparable to previously published data regarding patient age, underlying conditions, and surgical indications [[1, 2, 17, 22]. Further, our median FU of 7.5 years provides data comparable to other publications [[3, 11, 14, 16, 18, 20, 23].

### General considerations

The appendix offers several advantages, being a tubular structure, severed only at one communicating end, with almost independent blood supply [[25]. As stated above, we used the appendix whenever it was available and feasible; consequently, all but one patient underwent an appendicovesicostomy. Indeed, if the appendix is unsuitable or absent, Young and Monti described small intestine use as a possible substitute [26]. It requires, however, the bowel to be detubularized in order to fashion an adequately sized channel. This structure is therefore at increased risk of ischemia and stenosis. Also, the elasticity of the bowel wall unfortunately allows for pouch formation [[4, 24]. The use of the ureter, the Fallopian tube, and conduits created of bladder or stomach wall have also been described [[6].

Depending on the publishing team, different combinations are possible between appendix versus Monti and positioning in the right lower quadrant or in the umbilicus, making it difficult to compare our series to others. Of note, all the studies report a lower rate of complications when comparing appendix to segmental ileum use. Lefèvre et al. found Mitrofanoff complications in 45% of the patients in whom they used the appendix, versus 100% of Monti channels [[14]. Faure et al. had similar findings with all Monti, ureter, and colonic conduits presenting complications compared to 61% of appendix conduits [[13]. The appendix offers a suitable diameter for catheterization, with a relatively thick wall that directs the catheter well, and a distribution of vascularization along its entire length.

In our patient population, all but two CCC were placed in the right lower abdominal quadrant. While this position leaves the stoma more visible, conduit-skin anastomosis is made of scar-free tissue and can be as wide as deemed necessary, compared to a stoma positioned in the umbilicus. Physical considerations support right lower quadrant positioning: urine drainage will be performed in a position that allows for better spontaneous outflow of urine, as well as bladder irrigation in case of entero-cystoplasty. In contrast, drainage through the umbilicus requires an upward flow, potentially leading to incomplete emptying of the bladder and hindering mucus clearing, with increased risk of infection and bladder stone formation. Opinions on stoma positioning differ. Lefevre et al. found more complications in right lower quadrant stoma positioning (54%) vs umbilical positioning (25%), perhaps because a VQZ plasty is only used in 69% of right lower quadrant stomas [[14]. Leslie finds no difference in complications between a right lower quadrant stoma and an umbilical stoma [20]. For Faure, stenosis occurs more frequently in umbilical CCCs [13]. Overall, the choice of stoma positioning remains an issue of preferred style whereas VQZ plasty has been widely adopted. While the many differences in creating the CCC as well as positioning and fashioning the cutaneous stoma speak to the fact that not one single technique is clearly superior, the long-term course of our patients compared to other experiences supports our approach (Table 5).

**Table 5:** Data from literature review regarding continent catheterizable channels (CCC)

	Lefèvre 2018 [14]	Faure 2017 [13]	Kroll 2017 [3]	Welk 2008 [9]	Leslie 2010 [20]	Szymanski 2015 [16]	Jacobson 2017 [18]	Present study
Number of patients	34	54	62	54	169	204	74	31
Follow-up (m)	74	52	103	28	69	68	80	89
Appendix (%)	91	76	90	100	83	100	46	97
Location abdominal lower quadrant (%)	77	19	nd	0	26	nd	nd	94
VQZ plasty (%)	31	nd	nd	0	nd	nd	nd	94
General complication rate (%)	50	63	13 (if appendix)	19	39	18	47	19
CCC revision rate (%)	38	61	5	13	39	18	nd	13
CCC superficial stenoses (%)	9	31	nd	6	17	11	nd	0
CCC subfacial stenoses (%)	12	13	nd	2	8	6	nd	10
CCC incontinence (%)	4	17	5	6	10	2	4	3
CCC prolapsus (%)	nd	2	nd	6	4	nd	1	0
Time to first complication	Median 4 m	Median 25 m	nd	nd	nd	nd	Mean 24 m	Mean 37 m
Timing of complications	nd	84% in first 2 y	nd	80% in first 2 y	66% in first 3 y	69% in first 3 y 78% before 5 y	50% in first 2 y	57% in first 2 y
Latest post-op complication	nd	nd	nd	4 y	13 y	15 y	8 y	9 y

*m* months, *y* years, *nd* not defined

### Types of complications

As mentioned above, compared to the literature our CCC complication of 20% was rather low. Table 5 summarizes the recent literature in the field. When analyzing CCC using mainly the appendix as a conduit, the general complication rates vary between 12 and 63% [3, 9, 13, 14, 16, 18, 20]. According to the literature, most complications consist of catheterization difficulties or conduit incontinence. With regard to catheterization difficulties, publications detail superficial (cutaneous/supra-facial) or deep (sub-facial) stomal stenoses, including angulations, strictures, and false tracts.

*Superficial stenoses*: To create the stoma at the cutaneous level, direct suturing is the most straightforward approach [9]. Yet, to establish an easily accessible channel, spatulation of the conduit and creation of a V-flap sutured in the conduit is possible. This is believed to reduce the rate of cutaneous stomal stenosis. However, this technique has been linked to issues in relation to the exposed mucosa such as bleeding, mucus secretion, and a less than desirable cosmetic outcome [6, 10]. To overcome these drawbacks, the VQZ-plasty, first introduced by Ransley, adopts the advantages of the V-flap and covers the intestinal-cutaneous anastomosis with a skin flap, hereby concealing the stoma and protecting the mucosa [[10]. Reported rates of superficial stenoses vary between 6 and 31% [9, 13, 14, 16, 20]. None was observed in our study. Possible explanations might be in our surgical technique with wide conduit-to-skin anastomosis, a generous VQZ-plasty, and a protocol to keep an indwelling catheter for four weeks postoperatively, avoiding repetitive manipulation of the initially vulnerable tissue.

*Subfacial stenoses:* they are reported to occur at a rate of 2 to 13% [9, 13, 14, 16, 20]; in our study, we observed 10%, which situates us in good company.

*CCC incontinence:* this complication has been reported to occur at rates of 2–10% [3, 16, 18, 20], but can reach 17% or even 24% of patients, depending on the series [13, 14]. In our series we report a low rate of 3%, treated endoscopically. In the long-term survey results, none of the 21 patients reported this complication.

*CCC prolapsus*: they have been described in 1 to 6% of patients [9, 13, 16, 18, 20]. Again, in our study no patient was observed with this complication.

### Timing of complications

Secondary procedures were needed at 1, 12, and 20 months, then later at 4 and 6 years after the initial CCC surgery. It seems that the majority of CCC complications occur within the first two years after surgery. However, complications also may occur in the late postoperative period, with conservative management still being possible. These results are consistent with published data, reporting channel complications requiring surgical revision within 24 months [9, 18, 27]. Yet, Leslie et al. described a 30% stenosis rate in the first 3 postoperative years [20], and Szymanski mentioned the first 5 postoperative years as at increased risk for subfascial stenosis [16]. Indeed, complications are now increasingly described as occurring at any time, including in the long term, as the follow-up period increases. Jacobson et al. published in 2017 results that differ from their first analysis in 2006 with a shorter 28-month follow-up [28].

### Management of complications

Of the seven CCC dysfunctions, two cases of catheterization difficulties were managed conservatively. In both cases, mucosal edema played a significant role, resolving within 1 to 4 weeks of continuous drainage with an indwelling catheter. Two patients could be treated endoscopically. Only three patients, i.e., 10% of all patients, needed surgical revision, clearly below the reported rates, ranging from 38 to 61% [13, 14, 20]. Only Welk et al. have a similar 13% revision rate [9] and Kroll et al. with a 5% rate [3].

This said, as for the different management methods, some authors detail a “minimally invasive” management combining conservative ± endoscopic treatment, while others make subgroups between superficial surgery (supra-facial) and cystoscopy versus deep surgery (sub-facial). Thus, Faure et al. reported a conservative and endoscopic management rate of 82% [13], Welk et al. 44% [9], while in our series this rate is 57%. Complications have been reported to be managed surgically (open or endoscopic) in 60% of cases by Jacobson et al. [18] (including, however, Malone redo surgeries), 78% for Welk et al.[9], 77% for Lefèvre et al. [14] and 71% for our series.

### Short- and long-term surveillance

Our results show that our current surgical and nursing protocol results in very satisfying results. This said, we believe that the low complication rate observed during the FU period in our study can be attributed not only to the surgical techniques involving the appendix as a conduit and the VQZ plasty in the lower right abdomen, but also, undeniably, to the comprehensive nursing care provided. The constant and long-standing availability of our nursing staff, the ease with which patients can communicate with them regarding CIC difficulties, and their close involvement are critical factors that significantly contribute to the favorable outcomes reported. Not only do they provide practical hands-on instructions and tips on CIC, but the presence of this dedicated team also increases adherence of young people to their daily care and motivation of parents to support their child [29, 30]. Educational nurses also create a support system for the families, especially involving school nurses, to help them reach individualized goals step by step, to adapt to rhythms of family life, and to solve problems in day-to-day life. This team creates close links with patients, decreasing anxiety prior to the surgery, and following them during adolescence, supporting the transition from parental to child responsibility [31–34]. Indeed, in recent years specialized programs for children with complex medical conditions have emerged, and several studies published over the last decade report their significant positive impact on the care of both children and families [35, 36]. These programs not only improve long-term compliance but also reduce the use of emergency services and unplanned hospital admissions.

The value of long-term support, both medical and nursing, is reflected in the results of our follow-up questionnaire. The first significant result was the very high response rate of 91%, providing us with proof of good long-term compliance and adherence to treatment plans. In line with the literature [18, 20], all but one patient reported daily use of their CCC. With three to seven daily catheterizations via the CCC channel and a median FU of 7.5 years after stomal creation, catheterizations add up to 7800 to 18,650 tube passages per patient at the time of this study, in some patients considerably more. This calculation shows the extreme durability that is required in any CCC and, according to our results, durability seemingly met by the appendix, arguing again for its use.

Limitations of this study reside mainly in its retrospective nature. However, patients were followed up by the same nursing and surgical team throughout the study period, allowing for a very limited amount of missing data. Furthermore, the counterbalancing strength of this study lies in the consistency of the medical team, which remained unchanged throughout the duration of the research. This continuity ensured that both surgical procedures and postoperative management were standardized, providing a robust foundation for the reliability of our findings. Since a single operative technique was consistently applied, no internal control group was available. Therefore, we compared our findings with published experiences in similar patient populations.

## Conclusion

In conclusion, our findings support the use of the appendix in conjunction with VQZ plasty in the right lower quadrant for CCC procedures. Indeed, this study demonstrates a low revision rate for CCC dysfunction, with over half of the cases effectively managed by conservative or endoscopic means. It seems that following a stringent protocol with meticulous surgical technique and standardized postoperative care by a specialized nursing team reduces the risk of CCC complications. This combination of surgical precision and comprehensive care underscores the effectiveness of our approach in achieving favorable patient outcomes.

## Supplementary Information

Below is the link to the electronic supplementary material.

Supplementary file1 (PDF 50 KB)

## Data Availability

Data are provided within the manuscript and supplementary information files.

## Abbreviations

CCC: Continent catheterizable channel
CIC: Clean intermittent catheterization
FU: Follow-up
