# Supplementary material for: Mitrofanoff procedure in children: use of the appendix and VQZ plasty seems to minimize complications
Source: Pediatr Surg Int. 2025 Sep 26;41(1):304. doi: 10.1007/s00383-025-06204-6 (PMC12474596; doi:10.1007/s00383-025-06204-6)
Supplement: Supplementary file 1 — Supplementary file1 (PDF 50 KB) [file 383_2025_6204_MOESM1_ESM.pdf]

Mitrofanoff procedure in children : use of the appendix and VQZ plasty seems to minimize complications.

Adriana König, Ashley X. Wiseman, Barbara Wildhaber, Isabelle Vidal, Jacques Birraux
Corresponding author : isabelle.andrieuvidal@hug.ch

Items of the survey sent to the patients

1) Date of the last medical check-up for the vesicostomy.
2) Do you currently require any medical treatment for the bladder? No/ Yes. If yes, name of current medication.
3) Did you encounter any difficulties catheterizing the stoma (abnormally small passage)? No/ Yes. If yes: Date, and How was this situation managed by the medical team?
4) Did you encounter any urine leaks from the stoma? No/ Yes. If yes: Date, and How were the urine leaks managed?
5) Since the vesicostomy procedure, did you encounter any urine leaks through the natural channels? No/ Yes. If yes: Date, and How were the urine leaks managed?
6) Have you had other procedures for your vesicostomy? No/ Yes. If yes: For what reason did you require further intervention? What was the intervention?
1st intervention (Date/reason/ intervention). Possibility to complete answers until 4 interventions.
7) Do you still self-catheterize using your stoma? No/ Yes.
If no: What year did you stop?
If yes: How many times per day do you self-catheterize? What is the size of the catheter you use? Do you resort to continuous drainage during the night? No/Yes (hours per night).
8) Have you had one or more pregnancies? No/ Yes. If yes: number, C-section/ vaginal/ both (if multiple pregnancies).
9) Comments: Is there anything else you would like to share with us?
